# YY2 in Mouse Preimplantation Embryos and in Embryonic Stem Cells

**DOI:** 10.3390/cells10051123

**Published:** 2021-05-07

**Authors:** Raquel Pérez-Palacios, María Climent, Javier Santiago-Arcos, Sofía Macías-Redondo, Martin Klar, Pedro Muniesa, Jon Schoorlemmer

**Affiliations:** 1 Regenerative Medicine Program, Instituto Aragonés de Ciencias de la Salud, CIBA, Avenida San Juan Bosco 13, 50009 Zaragoza, Spain; rperezpa@unizar.es (R.P.-P.); smacir00@gmail.com (S.M.-R.); 2 Departamento de Anatomía, Embriología y Genética Animal, Facultad de Veterinaria, Universidad de Zaragoza, C/Miguel Servet 177, 50013 Zaragoza, Spain; mariacli@unizar.es (M.C.); fjsantiago@cicbiomagune.es (J.S.-A.); pmuniesa@unizar.es (P.M.); 3 Placental Pathophysiology and Fetal Programming Group, Fundación IISA, Avenida San Juan Bosco 13, 50009 Zaragoza, Spain; 4 Department of Neonatology, Charité—Universitätsmedizin Berlin, Augustenburger Platz 1, 13353 Berlin, Germany; martin_klar@web.de; 5 Fundación “Agencia Aragonesa para la Investigación y el Desarrollo” (ARAID), 50018 Zaragoza, Spain

**Keywords:** *Yin Yang 2*, YY2, preimplantation development, transposable elements, MERVL, mouse embryonic stem cells, self-renewal, *Zscan4*, expanded potency

## Abstract

*Yin Yang 2* encodes a mammalian-specific transcription factor (YY2) that shares high homology in the zinc finger region with both YY1 and REX1/ZFP42, encoded by the *Yin Yang 1* and *Reduced Expression Protein 1/Zinc Finger Protein 42* gene, respectively. In contrast to the well-established roles of the latter two in gene regulation, X chromosome inactivation and binding to specific transposable elements (TEs), much less is known about YY2, and its presence during mouse preimplantation development has not been described. As it has been reported that mouse embryonic stem cells (mESC) cannot be propagated in the absence of *Yy2*, the mechanistic understanding of how *Yy2* contributes to mESC maintenance remains only very partially characterized. We describe *Yy2* expression studies using RT-PCR and staining with a high-affinity polyclonal serum in mouse embryos and mESC. Although YY2 is expressed during preimplantation development, its presence appears dispensable for developmental progress in vitro until formation of the blastocyst. Attenuation of *Yy2* levels failed to alter either *Zscan4* levels in two-cell embryos or IAP and MERVL levels at later preimplantation stages. In contrast to previous claims that constitutively expressed shRNA against *Yy2* in mESC prohibited the propagation of mESC in culture, we obtained colonies generated from mESC with attenuated *Yy2* levels. Concomitant with a decreased number of undifferentiated colonies, *Yy2*-depleted mESC expressed higher levels of *Zscan4* but no differences in the expression of TEs or other pluripotency markers including *Sox2*, *Oct4*, *Nanog* and *Esrrb* were observed. These results confirm the contribution of *Yy2* to the maintenance of mouse embryonic stem cells and show the preimplantation expression of YY2. These functions are discussed in relation to mammalian-specific functions of YY1 and REX1.

## 1. Introduction

*Yin Yang 2* (*Yy2*) is one of a group of related genes encoding DNA-binding transcription factors that also includes *Reduced Expression Protein 1/Zinc Finger Protein 42* (*Rex1*/*Zfp42*) [1] and the prototype *Yin Yang 1* (*Yy1*) [2]. YY1 is a well-studied, ubiquitously expressed transcription factor with DNA-binding zinc fingers (reviewed in [3,4]). Dependent on the epigenetic environment and numerous interactions with chromatin regulators, including Polycomb Group complexes [5] and histone-modifying complexes [6], YY1 can function as an activator, repressor, or initiator of gene transcription. YY1 controls the expression of a wide variety of genes, through either classical promoter regulation, or regulation of enhancer–promoter interactions [7]. YY1 also participates in distinct mechanisms potentially related to oncogenesis [4], including increased mutagenesis, DNA repair, and chromosome segregation. Finally, YY1 contributes to silencing of an X chromosome (X chromosome inactivation (XCI)) [8]. YY1 function in XCI is essential for both normal expression of non-coding *Tsix* and *Xist* transcripts [9] and physical *Xist* association with the inactivation center [10].

While *Yy1* function has been demonstrated in a variety of tissues and developmental processes, *Yy1*-loss-of-function in the mouse also has revealed an essential function at the time of embryo implantation [11]. Mammalian embryonic development starts at fertilization, which is followed by formation of the zygote and cleavage divisions. Further development includes the successive generation of the morula and blastocyst, coinciding with the first lineage separation into inner cell mass (ICM) and trophectoderm, which contribute to the embryo and extra-embryonic tissues, respectively. The blastocyst hatches and starts the process of implantation in the uterus. Pluripotent, self-renewing embryonic stem cells (ESC) can be derived from the ICM of the preimplantation blastocyst. Maintenance of mouse ESC (mESC) in vitro depends on the core pluripotency factors *Oct4*, *Nanog*, and *Sox2* transcription factors, which form a cooperating network of transcription factors including also *Esrrb* and *Rex1*/*Zfp42* [12], which maintains a pluripotent signaling environment and epigenetic landscape.

The *Yy1* paralog *Rex1*/*Zfp42* is prominently expressed in embryonic stem cells and preimplantation embryos [1,13]. Modest levels of expression are also detected during spermatogenesis and early trophectoderm derivatives and placenta in the mouse [14] (Pérez-Palacios, Climent et al., in preparation). REX1 has been identified as a major regulator of *Tsix* expression [15] and XCI [16]. Depletion of *Rex1* in mESC does not affect expression levels of pluripotency-related transcription factors [15]. However, under conditions of *Rex1* deficiency, *Tsix* levels are reduced [15] and murine endogenous retrovirus-like (MERVL) expression [17] is induced, the latter both in mESC and during preimplantation development. MERVL is coregulated with several so-called two-cell stage genes including *Zscan4* [18], and there is also some evidence for regulation of *Zscan4* during preimplantation development by REX1 [13].

The *Yy2* gene most likely originates from a relatively recent retroposition event in an ancestor of placental mammals and evolved very quickly afterwards [19]. Expression of YY2/*Yy2* has been shown in most tissues and cell types investigated, including many adult organs [19,20], and in situ hybridization on mouse embryos showed ubiquitous *Yy2* expression from the 11.5 days post-coitum stage onwards [21]. Based on the similarity of in vitro binding sites and DNA-binding domains, both redundant and antagonistic functions for YY1 and YY2 have been proposed [22]. However, transcriptomics in depleted HeLa cells showed gene expression alterations specific to either YY1 or YY2 [23], and an analysis of a limited number of YY2 binding sites in mouse trophectoderm stem (TS) cells showed no YY1 binding to these sites in vivo [24]. Recently, identification of target genes by ChIP-Seq assay in mESC [25] showed that the majority of YY2 targets are non-overlapping with YY1 targets [26,27]. Altogether, the combined data support that YY1 and YY2 mostly regulate unique sets of genes. A combination of ChIPseq data and expression data in embryoid bodies (EB) derived from YY2-overexpressing cells suggests a role for YY2 as an activator of transcription in differentiation towards a cardiovascular lineage [25]. In mESC, *Yy2* expression levels are controlled by splicing-dependent differential translation. The resulting stringent regulation of *Yy2* expression levels is critical for proper mESC self-renewal and lineage commitment [25].

Repeat DNA and transposable elements (TEs) including endogenous retroviruses (ERVs) occupy >50% of mammalian genomes [28,29]. While they contribute to biological processes, their expression is generally considered deleterious to normal physiology and genome integrity [30,31]. Specific TEs including LINE, Intracisternal A-particle (IAP), and MERVL are expressed in a stage-specific pattern in both human and mouse preimplantation embryos [31,32]. Altering normal levels of either LINE-1 [33] or MERVL-containing transcripts [34] during preimplantation stages interferes with normal developmental progress, probably as a result of altered chromatin accessibility [35]. The expression of MERVL in mESC is associated with expanded potency, attributed to so-called two-cell-like cells [32,36]. While sequences related to the YY1-binding motif element are enriched at LINE, IAP, and MERVL elements, and YY2 binding has been reported [24,28], the extent to which YY1 or its paralog YY2 contribute to this regulation during preimplantation development and in mESC has not been extensively studied.

Despite the presence of both YY1 and REX1 in preimplantation embryos [1,13], neither the presence of YY2 at preimplantation stages nor a functional role for *Yy2* at stages before blastocyst outgrowth/implantation has been previously addressed. This functional role may reside in the regulation of either *Zscan4* [18], as described for REX1 [13], or transposable elements. YY2 binding to the latter has been reported in mESC [24] and TS cells [17], especially to MERVL and IAP. However, as opposed to the regulation of these elements by REX1 in early embryonic stages [17], similar regulation by YY2 has not been previously addressed. It was previously shown that propagation of mESC requires *Yy2* [25]. However, in the absence of viable *Yy2*-deprived cells, gene expression analysis in mESCs with attenuated *Yy2* levels was very limited and did not define the role of *Yy2* in ES self-renewal and pluripotency. Therefore, while YY2 binding sites in mESC were identified, results on the potential deregulation of pluripotency genes were mostly derived from YY2 overexpression studies. Moreover, deregulation of transposable elements in mESC as a function of *Yy2* levels was not addressed at all [25].

In the present manuscript, we first set out to show the presence of YY2 in preimplantation embryos from zygote onwards. Once its presence was established, we addressed both whether attenuation of *Yy2* levels might alter preimplantation development in vitro and the possibility that a lack of YY2 may deregulate the expression of co-regulated *Zscan4* and MERVL, as well as IAP elements. Furthermore, in an independent effort to confirm and analyze *Yy2* function in mESC, we studied mESC with attenuated *Yy2* levels. For the first time, we addressed both colony formation capacities and pluripotency-associated gene expression in these cells. Finally, we were then able to test whether YY2 binding to MERVL and IAP elements in ES cells is translated into deregulation of such elements in the absence of YY2.

## 2. Materials and Methods

### 2.1. Oocyte and Embryo Collection and In Vitro Culture

Oocyte and embryo collection were performed according to standard procedures [37]. Four- to six-week-old females were superovulated by an intraperitoneal (i.p.) injection of pregnant mare serum gonadotropin (PMSG, 5 IU) followed by human chorionic gonadotropin (hCG; 7.5 IU) 48 h later. Fertilized eggs were harvested at 20 h post-hCG in M2 media (Sigma-Aldrich, Ref. M7167, St. Louis, MO, USA. Cumulus cells were removed by thorough pipetting in M2 media containing hyaluronidase. Good-quality zygotes were thoroughly washed three times in clean dishes containing M2 media and KSOM (Embryomax^®^; Millipore, Ref. MR-020P, Burlington, MA, USA, and cultured in vitro in KSOM at 37 °C, 5% CO_2_, 90% relative humidity.

All procedures were carried out under Project License PI29/08, approved by the in-house Ethic Committee for Animal Experiments from the University of Zaragoza. The care and use of animals were performed according to the Spanish Policy for Animal Protection RD1201/05, which meets the European Union Directive 86/609 on the protection of animals used for experimental and other scientific purposes.

### 2.2. Immunofluorescence and Confocal Microscopy

Mouse embryos (CD-1, Charles River, Lyon, France) for immunostaining were obtained from natural matings using standard methods. Immunostaining and confocal microscopy were routinely performed as previously described [13,38,39], with minor modifications. Briefly, mouse embryos from natural matings at the indicated stage were washed in PBS for 5 min, fixed at room temperature (RT) for 20 min in 2.5% paraformaldehyde in PBS (pH 7.4), and permeabilized with 0.2% Triton-X100 in PBS for 20 min at RT. Embryos were then blocked/permeabilized in PBS/0.2% Triton-X100/10% FBS for 1 h at RT. All the following incubations and washes were performed in PBS/0.2%Tx100/2% FBS (PTF). Fixed and permeabilized embryos were then incubated overnight (o.n.) at 4 °C with primary antibodies. Anti-YY2 antibody was used at (1:4800) dilution. Embryos were washed three times for 15 min and incubated with anti-rabbit biotin (1:150) secondary antibody for 1h at RT. Embryos were washed three times for 15 min and incubated with streptavidin-488 (1:400) for 1h at RT, followed by three washing steps in the dark. Nuclei were counterstained with DAPI (10 μg/mL) in PBS for 20 min. Embryos were mounted in glycerol/DABCO 2.5%/Tris pH8.6 (DTG). Negative controls were performed using the same procedure without the addition of primary antibody. Confocal sections were obtained using an Olympus confocal microscope Fluoview FV1000 with 40X objective (Olympus Europe, Hamburg, Germany). Images were pseudocolored as follows: YY2 in green and DAPI in blue (cell nuclei).

### 2.3. Immunological Reagents and Western Blot

A rabbit antiserum raised in house against a GST-YY2Δ protein (amino acids 1 to 173) and IgG-purified according to standard methods has been described in detail elsewhere [24]. To prove specificity for YY2, the serum has previously been tested in Western blot and immunoprecipitation assays, comparing HA-tagged YY2 with HA-tagged YY1 in 293T cells (Figure S3 in [24]). The experiments demonstrated that this antibody is specific for YY2, showing also absence of binding to YY1, absence of IF staining in non-transfected 293T cells, and absence of cross-reaction with YY1 in transfected 293T cells. Monoclonal α-HA (clone HA-7) was obtained as an unpurified ascites fraction (Sigma H9658). Preparation of cell lysates, separation by SDS-PAGE, and Western blot were carried out as described [17]; equivalent loading in each lane is demonstrated by reprobing the membrane with an αTUBULIN antibody.

### 2.4. Embryo Microinjection with siRNAs

Zygotes were collected from superovulated B6D2F1/J females mated to B6D2F1/J males (Charles River, Lyon, France) at 20 h post-hCG (defined as Day 1), maintained in M2 media, and microinjected into the cytoplasm with siRNA duplexes (100 μM). Gene-specific Stealth^TM^ siRNA duplexes were purchased from Invitrogen and prepared as described [13]. Sequences are listed in Table S2; Stealth™ RNAi Negative Control Med GC (Life Technologies Corporation, Ref. 12935300, Carlsbad, CA, USA) was used as a control.

Texas Red detection was used to monitor siRNA microinjections. Degenerated embryos were removed both immediately after injection and 18 h after. The number of embryos and their developmental stage (e.g., number of cells) were evaluated daily. Images were captured using a Leica DFC360Fx camera adapted to a Leica M165FC stereomicroscope (Leica Microsystems AG, Heerbrugg, Switzerland) using Leica Application Suite 3.2.0 (Leica Microsystems Ltd., Heerbrugg, Switzerland). For subsequent gene expression analysis, embryos were harvested at the indicated morphology in TRIzol^®^ (Life Technologies Corporation, Carlsbad, CA, USA), flash-frozen, and stored at −80 °C.

### 2.5. Gene Expression in Preimplantation Embryos

Embryos were harvested at 1-cell, 2-cell, 4-cell, 8-cell, morula, blastocyst, and late blastocyst stage, at 20, 43, 55, 66, 80, 92 h post-hCG, and at embryonic day 4.5, respectively. Oocytes were collected at 14 h post-hCG from unmated females. An identical number of experimental and control oocytes or embryos from the same experiment (generally 8 to 10 embryos) were pooled and homogenized in 100 μL TRIzol^®^. Total RNA from embryos was purified by a two-round extraction protocol. RNA was firstly isolated from TRIzol^®^ by chloroform extraction, ethanol precipitation using glycogen (Roche, Basel, Switzerland) as a carrier, pelleted by centrifugation, and re-suspended in DEPC-treated water. RNA was further DNAse-treated with RQ1 RNase-free DNase (Promega), phenol extracted, re-precipitated with ethanol, and re-suspended in DEPC-treated water. Reverse transcription of purified RNA was performed using the ThermoScript^®^ RT-PCR System (Life Technologies Corporation, Carlsbad, CA, USA) with random hexamer primers. cDNA from one to two oocyte or embryo equivalents were used as a template for each PCR reaction, and amplified using the following PCR program: 35 cycles of denaturation at 94 °C for 30 s, 30 s of annealing and 1 min extension at 72 °C; and a final 5 min extension. PCR products were visualized using ethidium bromide and photographed on a Gel Doc transilluminator (BIO-RAD, BIO-RAD Laboratories, Segrate, Milan, Italy). Primers are listed in Table S2.

### 2.6. Cell Culture, shRNA Knockdown, and RT-qPCR Analysis

Procedures for tissue culture of mESC line E14T [40], colony formation assays after shRNA-mediated knockdown, and RT-qPCR analysis were carried out as described previously [17]. mESC were cultured in in the presence of 10% fetal calf serum, leukemia inhibitory factor (LIF), and 2i as described [41], on gelatin-coated tissue culture dishes. Vectors used for the episomal expression of short hairpin RNAs (shRNAs) [17] contained sequences for episomal maintenance, PGKHygro, a H1 promoter [42], and short hairpin sequences, which were designed using specialized software [43]. Two different sequences were inserted: 5′ CAATACCACTCTCCTGTTATT as sh1*Yy2*; 5′ GTAGTAGAGATCATGATAA as sh2*Yy2*; a GFP sequence was used as a control: 5′ AAGCGCGATCACATGGTCCTG.

For the subsequent evaluation of colony formation assays (colony numbers, protein extraction, and RNA extraction), colonies grown in the presence of selective agent for 7 days were carefully washed twice with ice-cold PBS (avoiding colony cell loss). For colony numbers and self-renewal analyses, cell colonies were fixed at 4 °C in 80% ethanol. Posterior analysis included staining for alkaline phosphatase (AP) (Sigma-Aldrich, Ref. 85L3R, St. Louis, MO, USA) and counting of colonies (Nikon Diaphot Inverted Tissue Culture Microscope, (Nikon Corporation, Tokio, Japan)). For each condition, ten fields of predetermined size were counted; colony numbers of three independent experiments are represented as mean ± standard deviation. Pearson’s chi-squared test was subsequently used to determine whether the difference between the frequencies observed after treatment with each shRNA were statistically significant. Alternatively, cells were directly harvested in TRIzol^®^ or in protein lysis buffer for further gene expression (RT-qPCR) and protein level (Western blot) analyses.

Attenuation of *Yy2* levels with a third hairpin (5′ TTCAGTCCTGAATTTGGAAGC named sh3*Yy2*) was studied after transient transfection. The construct contained a CAGG-driven EGFP and an H1 promoter for expression of hairpin RNA. Two days after transfection, cells were sorted to separate GFP+ from GFP- cells, which were subsequently lysed in TRIzol^®^ for posterior gene expression analysis.

Gene expression was analyzed by quantitative PCR; all primers are listed in Table S2. Expression levels were normalized to *Gapdh* as a reference gene and depicted as fold expression (2^−∆∆Ct^) [44] relative to mESC-expressing shControl RNAs. The origin and specificity of TE primers has been amply discussed [17]. Statistical analyses were performed using GraphPad software (San Diego, California USA, software version 9.0.2. for MacOS).

## 3. Results

### 3.1. Expression of YY2 during Preimplantation Development

A publicly available database of expressed-sequence tags (ESTs) (Unigene database) was searched for ESTs encoding *Yy2*. While ESTs for diverse adult body parts including bone marrow, bone, lung, mammary gland, skin, spleen, testis, and thymus were listed in the database, no pancreas, eye, or intestine-derived ESTs have been reported. We also found that among 31 cDNA *Yy2* clones described, no EST was detected in oocytes or embryos at the zygotic stage, but several originated from libraries of embryos at cleavage, morula, and blastocyst stages (Figure S1). This in silico analysis indicates that *Yy2* is expressed during embryonic preimplantation development.

**Figure 1:**
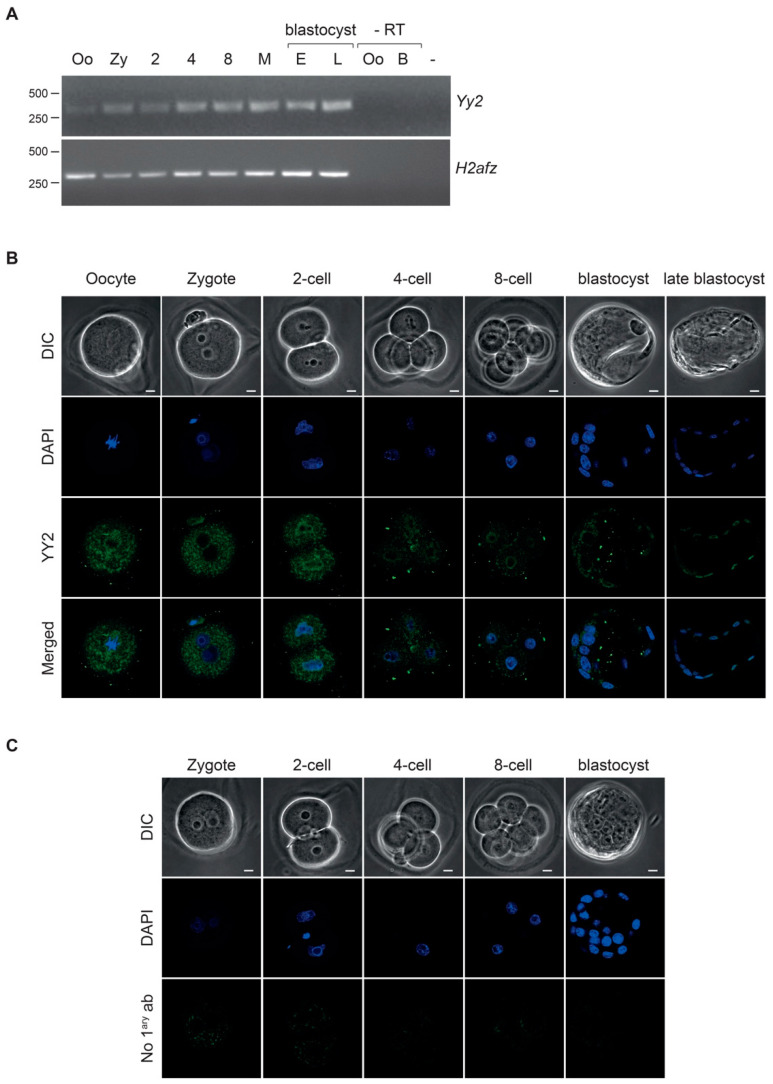
YY2 is present throughout mouse preimplantation development. (**A**) Detection of mRNA encoding *Yy2* in mouse oocytes and preimplantation embryos by RT-PCR. RNA extracted from ~ 25 pooled oocytes or embryos at each stage was reverse-transcribed and subjected to PCR using primers to amplify either *Yy2* or *H2afz* gene as a control. O, oocyte at metaphase II, Zy, zygote; 2, 2-cell; 4, 4-cell; 8, 8-cell embryos; M, morula, E, early blastocyst, L, late blastocyst (E4.5). RT refers to controls without reverse transcription; a PCR reaction without input (*-*) is shown to the right. The sizes of molecular weight markers run alongside are indicated on the left. (**B**) Indirect immunofluorescent detection of YY2 in confocal sections of representative mouse embryos depicted as DIC images. DAPI staining indicates chromatin (blue). The antibody used has been shown to specifically detect YY2 in immunofluorescence (Figure 1A and Figure S4 in [24]). Scale bar, 10 µm. (**C**) Absence of unspecific background in indirect immunofluorescence. Confocal sections of preimplantation mouse embryos incubated without primary antibody (control), processed in parallel to the indirect immunofluorescent detection of YY2 shown in Figure 1B. Scale bar, 10 µm.

To experimentally confirm the expression patterns of *Yy2* during early development, we performed RT-PCR analysis of pooled oocytes and embryos at preimplantation stages. *Yy2* mRNA was detected in oocytes and early embryos at all developmental stages (Figure 1A). Surprisingly, in contrast with the in silico prediction, *Yy2* was expressed in oocytes and zygotes, although its levels of expression were lower than in blastocysts (Figure 1A, Figure S1B). From the two-cell stage onwards, *Yy2* mRNA levels gradually increased, reaching peak levels in the late blastocyst.

To study the temporal and spatial expression pattern of the YY2 protein, we performed indirect immunofluorescence analyses using an affinity-purified polyclonal serum, raised against the aminoterminus of YY2 [24]. The specificity of this serum for YY2 has been extensively confirmed previously; see Materials and Methods for a full description of relevant data. In accordance with the presence of *Yy2* transcripts (Figure 1A), immunostaining revealed YY2 expression at all preimplantation stages tested, showing a more prominent staining in two-cell embryos, which may correspond to either increased protein levels or nuclear clustering (Figure 1B). Starting at the one-cell stage until the blastocyst stage, anti-YY2 immunoreactivity was detected in all cells, although dynamic subcellular localization patterns were evident. YY2 staining was detected in the nucleus at all embryonic stages analyzed, although some potential cytoplasmic localization might be observed in embryonic cells and metaphase II (MII) oocytes above the antibody unspecific background (Figure 1B,C). In embryonic day 3.5 (E3.5) blastocysts (Figure 1B), YY2 showed a mixed perinuclear–cytoplasmic localization pattern in the ICM compartment, while nuclear staining was observed in cells within the trophectoderm. YY2 staining became progressively more nuclear in trophectoderm cells at the late blastocyst stage. YY2 showed dynamic levels of expression during preimplantation, with apparent fluctuations in expression level and intracellular localization.

### 3.2. In Vitro Development of Yy2 Depleted Embryos

**Figure 2:**
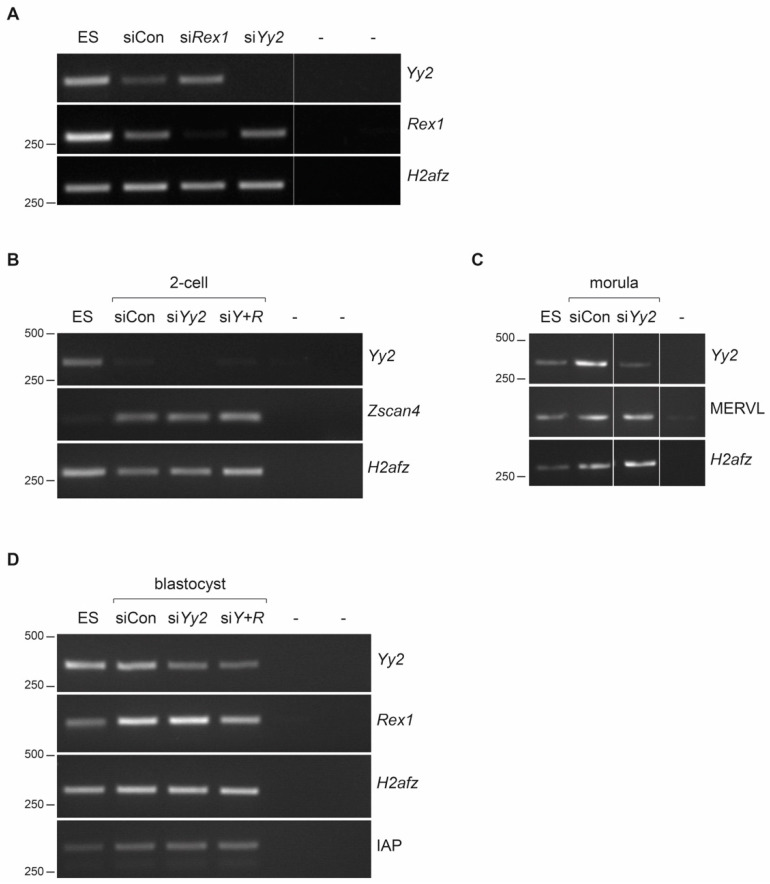
Attenuation of *Yy2* levels in mouse preimplantation embryos. (**A**) *Yy2* and *Rex1* levels in siRNA-injected embryos. mRNA levels were assessed by semi-quantitative RT-PCR analysis in embryos at the morula stage injected with either control siRNA (siCon), *Rex1* siRNA (si*Rex1*), or *Yy2* siRNA (si*Yy2*); mESC RNA was used as a positive control. PCR reactions without input (-) did not yield any products. *H2afz* was used as a control. The sizes of molecular weight (Mw) markers run alongside are indicated on the left. (**B**) Developmental progress assessment of *Yy2*-depleted preimplantation embryos. Injections of control siRNAs (siControl) and siRNAs against *Yy2* (si*Yy2*) were performed in parallel and embryonic development was monitored daily. The ratio of the number of embryos at the specified stage over the total number of embryos, and the average percentage, is indicated. The day of zygotic microinjection was set as Day 1. Cumulative data of three independent experiments are presented, except for data at Day 5, which corresponds to 1 and 2 experiments for siControl and si*Yy2*, respectively. A technical issue for the control condition on Day 5 precluded normal developmental progression; therefore, Day 5 data of this condition are limited to only one experiment (see Table S1). (**B**–**D**) Effect of attenuation of *Yy2* levels on gene expression. One-cell embryos were microinjected with control siRNAs (siCon), *Yy2*-specific siRNAs (si*Yy2*), or a combination of *Rex1* and *Yy2* siRNAs (siY + R). Embryos were cultured in vitro to the 2-cell (**B**), morula (**C**), or blastocyst (**D**) stages. RNA was extracted, reverse-transcribed, and gene expression of equivalent amounts of embryos was analyzed by semiquantitative RT-PCR with primers specific for the indicated genes; RNA extracted from mESC was processed alongside. *H2afz* was used as a control. (-) refers to PCR reactions without template added. The sizes of Mw markers run alongside are indicated on the left.

To attenuate *Yy2* expression during preimplantation development, we designed siRNAs (Stealth^TM^ RNAs; see Materials and Methods). The efficiency of siRNAs was tested by co-transfection with HA-YY2 in 293T cells. Expression of HA-YY2 was abolished by co-transfection of S5 Stealth RNAs and, to a much lesser degree, by S6 Stealth RNAs, but not by control siRNAs (Figure S2A). Efficiency of these *Yy2* S5 Stealth RNAs (si*Yy2* hereafter) and siRNA against *Rex1* (si*Rex1*) in mouse embryos was assessed after injection of one-cell embryos. *Yy2* and *Rex1* mRNA levels were monitored in injected embryos at the morula stage. Expression of *Rex1* was severely suppressed in the si*Rex1*-injected embryos (Figure 2A). The converse was true in si*Yy2*-injected embryos, which displayed unaltered levels of *Rex1* and attenuated levels of *Yy2*. No changes were detected in the expression of the *H2afz* gene, used as a control.

In order to examine whether transient depletion of *Yy2* affected early embryonic development, one-cell embryos were collected and injected with control siRNAs (nonsense oligonucleotides) or with si*Yy2* and cultured in vitro. Similar numbers of embryos were injected with either *Yy2* or control siRNAs in each experiment, and successful injection was visualized using co-injected Texas Red (Figure S2B). Two-cell embryos were selected for further culture and developmental progress was examined daily. After injection of 104 and 114 embryos in total (siControl and si*Yy2*, respectively) on three independent experiments, we were unable to observe differences in developmental progress between embryos injected with control siRNAs or siRNAs directed against *Yy2* (Table 1, Table S1). Control and si*Yy2*-injected embryos developed into two-cell embryos (100 and 99.1%, respectively; day 2), 5–8-cell embryos (88 and 82.2%, respectively; day 3), morulae (90 and 93%, respectively; day 4), and blastocysts (80.8 and 76.3%, respectively; day 5) with equal efficiency (Table 1, Figure S2B). No statistically significant differences were found between the two groups at the stages analyzed.

Given the absence of a phenotype in *Yy2*-depleted embryos, we addressed the effect of simultaneous depletion of both *Yy2* and *Rex1*. Similar injection experiments were performed, resulting in no significant developmental defects or delays (Supplementary Table S1). Similar to control embryos, the doubly depleted embryos developed with high efficiency into 5–8-cell embryos (95%), morulae (92%), and blastocyst stages (82%) on consecutive days.

### 3.3. Yy2 Loss-of-Function Mouse Embryos Express Normal Levels of ERV Elements

We assayed the gene expression of the embryo-specific gene *Zscan4* and selected transposable elements by RT-PCR at different developmental stages, in embryos with attenuated *Yy2* levels, which were generated using siRNAs as described above. While weak but detectable expression of *Yy2* in two-cell embryos seemed slightly attenuated by the siRNAs (Figure 2B), we could not detect differences in the levels of *Zscan4* expression. Additionally, under these conditions, neither MERVL expression at the morula stage (Figure 2C) nor the blastocyst-specific expression of IAP elements was altered (Figure 2D). IAP expression was also not affected in *Yy2*+*Rex1* doubly depleted embryos at the blastocyst stage. Altogether, we found no evidence for altered gene expression as a result of *Yy2* depletion in preimplantation embryos.

### 3.4. Depletion of Yy2 Expression Alters Self-Renewal in Mouse ES Cells

**Figure 3:**
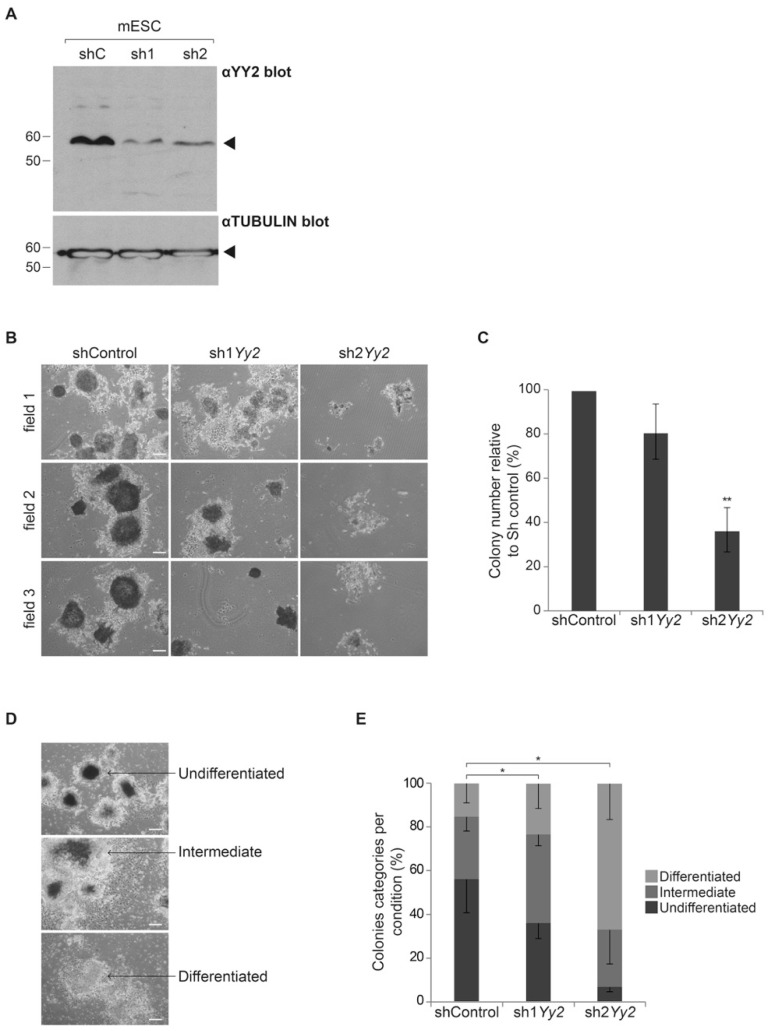
Attenuation of *Yy2* levels impacts on colony formation capacity in mESC. (**A**) Western blot to detect YY2 (Top) or TUBULIN (Bottom) in cell lysates of mESC transfected with plasmids that express either control shRNAs (shC) or shRNAs directed against *Yy2*, sh1*Yy2* (sh1), and sh2*Yy2* (sh2). Migration of molecular weight standards is indicated to the right; detected proteins are marked with arrowheads. (**B**–**E**) Clonogenic colony formation assays. Equal numbers of mESC were transfected with shRNA-expressing plasmids, shControl, sh1*Yy2*, or sh*Yy2*, and were selected for 7 days with hygromycin. (**B**) The resulting colonies were stained for alkaline phosphatase (AP) and photographed. Three representative fields are shown for each condition. Scale bar, 100 µm. (**C**) Quantification of the total number of colonies obtained in (**B**). Colonies per field (10 fields) were counted; the number of colonies formed by cells expressing control shRNA was set at a 100% and the number of colonies in the presence of shRNAs directed against *Yy2* was calculated accordingly. Error bars represent the standard deviation over three independent assays. ** *p*-value ≤ 0.01 (one sample *t*-test). (**D**,**E**) Colony morphology assessment. Quantification of colonies according to AP staining and colony morphology obtained in (**B**). Colonies were categorized as pluripotent, intermediate, and differentiated according to the examples indicated. Scale bar, 100 µm. (**E**) Total number of colonies of cells in the shControl, sh1*Yy2*, and sh2*Yy2* conditions in a predetermined number of fields (10 fields) was counted and classified in each category. The relative percentage of each category per condition is represented as a stacked bar graph. Error bars represent the standard deviation over three independent experiments. * *p*-value < 0.001 (X^2^ test).

To attenuate *Yy2* expression in mESC, we designed two hairpin constructs, termed Sh1*Yy2* and Sh2*Yy2*. YY2 protein levels in mESC were severely reduced in cultures over-expressing either hairpin RNA, as opposed to cells expressing the control shRNA (Figure 3A). We subsequently investigated *Yy2* function in mESC by using stable episomal expression of these shRNAs. Cells were electroporated and selected for expression of Hygromicine resistance co-carried on the shRNA plasmids, seeded at clonal densities, and colony formation was analyzed as a measure of self-renewal. Cells expressing shRNAs targeting *Yy2* formed fewer colonies compared to cells electroporated with control shRNAs (Figure 3B), accounting for 81% and 37% of the total number of colonies in Sh1*Yy2* and Sh2*Yy2* conditions, respectively, relative to the total number of colonies in shControl (Figure 3C). ES colonies were classified into three different categories based on morphology and alkaline phosphatase (AP) staining: undifferentiated—colonies with smooth edges and rounded shape, limited presence of differentiated cells and strong AP positive staining; intermediate colonies—those with irregular edges, considerable number of differentiated cells, and faint AP staining; and differentiated colonies—with no AP positive staining. Colony morphology was affected by *Yy2* depletion, as the number of undifferentiated colonies diminished 1.5- and 8.8-fold in colonies expressing sh1*Yy2* and sh2*Yy2*, respectively (Figure 3D). In contrast, the percentage of colonies with either an intermediate or differentiated morphology increased in both conditions (3- and 7.3-fold for sh1*Yy2* and sh2*Yy2*, respectively (Figure 3D)). The observed difference in colony morphology was significant between control and both sh1*Yy2* (X^2^ = 106.64, df = 2, *p*-value < 0.05) and sh2*Yy2* (X^2^ = 604.9, df = 2, *p*-value < 0.001). Altogether, these data show that reduced expression of *Yy2* was coincident with reduced self-renewal in mESC.

### 3.5. Gene Expression in Yy2-Depleted ES Cells

**Figure 4:**
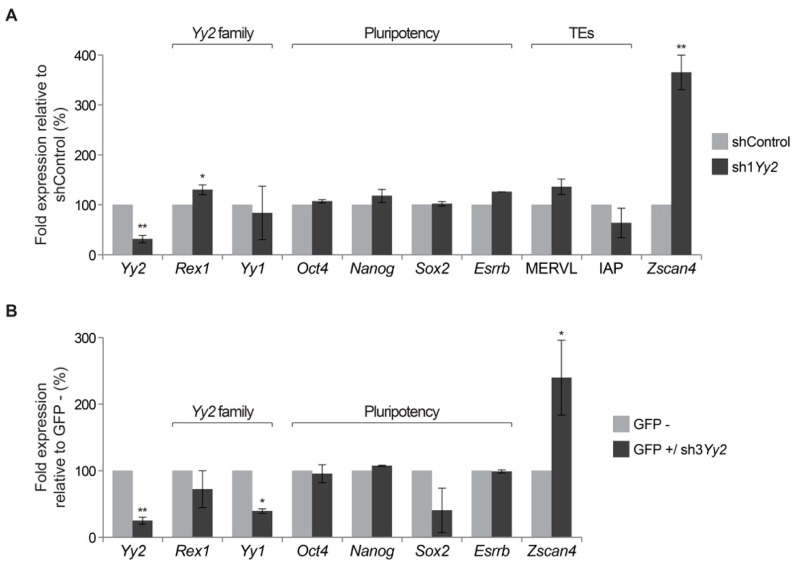
Gene expression in *Yy2*-depleted mESC. (**A**) Gene expression in mESC colonies transfected with either sh1*Yy2*- or shCon-expressing plasmids was measured by qPCR. Expression levels are depicted as fold expression in depleted cells relative to mESC-expressing shControl. Data presented for sh1*Yy2* are the mean of 3 biological replicates, each performed on different days and using a different preparation of cells (except for *Yy1* and *Esrrb* data that correspond to two replicates and one experiment, respectively), and three technical replicates for each experiment. The statistical significance (one sample *t*-test) of differences in gene expression is indicated, ** *p*-value ≤ 0.01, * *p*-value ≤ 0.05; all other differences were not significant. (**B**) Expression levels of *Yy2* gene family members, pluripotency markers, and *Zscan4*, measured in cells transfected with a GFP-expressing sh3*Yy2* construct. Cells were sorted based on GFP expression, and expression is depicted as fold expression of GFP+ relative to GFP- cells. Data shown are based on technical replicates. ** *p*-value ≤ 0.01, * *p*-value ≤ 0.05 (one sample *t*-test).

To further understand the molecular changes associated with the depletion of YY2 in mESC, we extracted RNA from *Yy2*-depleted colonies (and controls) and performed RT-qPCR gene expression analyses. We first compared the expression levels of pluripotency factors in the colonies analyzed, to mESCs obtained from regular culture. Results (Figure S3) indicate that the control colonies (shControl) express levels of core pluripotency factors comparable to mESC. We therefore assume that the cells making up these colonies can be considered mostly undifferentiated as expected, although some differentiation is apparent in the colonies after 7 days of culture (Figure 3B). We then analyzed whether changes in the levels of *Yy2* (Figure 4A) could affect the expression of other members of the transcription factor family. In *Yy2*-depleted cells, *Rex1* levels appeared slightly but significantly increased (*p*-value ≤ 0.05), while *Yy1* levels were not changed, although a trend of downregulation and some variability was observed between independent experiments (Figure 4A). We next measured the expression of several pluripotency markers and no significant differences of expression were detected for *Oct4*/*Pou5f1*, *Sox2*, *Nanog*, and *Esrrb* mRNAs as a result of attenuation of *Yy2* levels (Figure 4A, Figure S3A). Using primers that amplify regions conserved among several copies of transposable elements, we did not detect significant differences in the expression levels of either MERVL or IAP (Figure 4A). We finally investigated the expression of *Zscan4* in *Yy2*-depleted cells. We detected a significant induction of *Zscan4* levels in *Yy2*-depleted cells as opposed to control cells (3.6-fold change, *p*-value ≤ 0.01, Figure 4A).

To validate these results, we transfected mESCs with an shRNA against *Yy2* containing an EGFP cassette (sh3*Yy2*) and compared the levels of gene expression of GFP-expressing (GFP+) versus GFP-non-expressing (GFP-) cells by RT-qPCR. As expected, GFP + sorted cells had lower levels of *Yy2* compared to GFP-cells (Figure 4B). Similarly to the results obtained in the previous approach, *Yy2* family members were somewhat affected in *Yy2*-depleted cells GFP + cells. While no significant changes were observed for *Rex1*, *Yy1* mRNA levels were significantly downregulated. Consistent with the results shown in Figure 4A, no significant changes in gene expression levels were detected in the pluripotency markers analyzed (*Oct4*/*Pou5f1*, *Sox2*, *Nanog*, and *Esrrb*), although the level of *Sox2* trended downwards. As before, the *Yy2*-depleted GFP + cells showed a 2.4-fold increase in *Zscan4* levels compared to GFP- cells (Figure 4B). From the combined experiments, we conclude that YY2 contributes to the regulation of *Zscan4* levels in mESC.

## 4. Discussion

The data on YY2/*Yy2* expression in time and space are by no means complete. We had previously shown that YY2 protein is expressed in mouse ES and TS cells [24]. We now report the presence of *Yy2* mRNA and protein starting in oocytes and in preimplantation embryos at all stages. We show that YY2 is present during all stages of preimplantation development, and dynamically localized between the nucleus and cytoplasm. As opposed to a steady and gradually increasing expression of *Yy2* throughout preimplantation development, we detected apparently higher levels of YY2 specifically at the two-cell stage, showing clear nuclear expression above the cytoplasmic staining. This pattern may be indicative of a potential clustering of YY2 protein (and associated functional relevance at these particular stages) or directly result from higher expression levels. The latter case is compatible with a non-linear relationship between mRNA and protein levels described in mESC [25]. While knockdown of *Yy2* mRNA was clearly achieved, development until blastocyst under these conditions proceeded in the absence of temporal or morphological alterations. While depletion of *Yy2* has been shown to interfere with blastocyst outgrowth [25], we find no evidence for an earlier role in vivo according to morphological and viability criteria. However, more subtle defects cannot be ruled out and defects may have been further masked by the continued presence of protein from maternal deposits (see the presence of *Yy2* mRNA and YY2 protein in the oocyte, Figure 1). Furthermore, implantation and posterior development of either the embryos or extra-embryonic tissues is likely to depend on YY2, as *Yy2* depletion interferes with blastocyst outgrowth in vitro [25]. The continued presence of *Yy2* mRNA and protein during preimplantation embryos at all stages overlaps with the YY1 transcription factor [45] and ZFP42/REX1 [13]. As *Yy1* and *Rex1* contribute to mammalian-specific processes such as regulation of transposable elements and XCI, contributions of *Yy2* to these processes may still be envisaged.

YY2 shares homology in the DNA-binding zinc fingers with both YY1 and ZFP42/REX1. Despite a high degree of homology in the DNA-binding domain between YY1 and YY2, target genes for both genes are mostly unique to either of them [24,25], although some overlap exists [23,25]. Certainly, overlap exists to some extent in YY1 and YY2 binding to specific TE [17,46] and YY2 associates with MERVL and IAP elements in mESC [24]. In contrast to previously reported regulation of IAP and MERVL elements by YY1 [46] and REX1 [17], respectively, we were unable to detect altered expression of these elements in mouse embryos or mESC with attenuated levels of *Yy2*. However, similar to YY1, YY2 may interact with the TRIM28-mediated silencing machinery through LSD1 [18,32]. Therefore, *Yy2* may regulate the expression of other TEs, at stages not investigated, or be supplanted by YY1 or REX1 in its absence.

It was previously shown that propagation of mESC requires *Yy2* [25]. However, in the absence of viable *Yy2*-deprived cells, data presented in this report did not define the role of *Yy2* in ES self-renewal and pluripotency. Careful titration of selective agent and the use of episomal vectors allowed us to obtain ES colonies with constitutively diminished *Yy2* levels. Reduced expression of *Yy2* in mESC compromised their colony-forming capacities, resulting in lower numbers of colonies and more differentiated cells (Figure 3). Surprisingly, we failed to detect alterations in the expression levels of a set of pluripotency-associated genes including *Sox2*, *Oct4*, *Esrrb*, and *Nanog* (Figure 4A, Figure S3). This result was repeated when analyzing cells sorted for sh3*Yy2* expression, with the exception of a weak downregulation of *Sox2* levels (Figure 4B). Tahmasebi et al. have argued that the essential function of YY2 in mESC is most likely mediated by direct transcriptional regulation of ESC genes that they identified in genome-wide YY2 binding studies, which include *Oct4*, *Esrrb*, *Eras*, *Tet1*, *Tet2*, and *Tdgf1* [25]. This proposal was further based on results of YY2 overexpression studies and attenuation studies in which transient high overexpression of *Yy1* was also observed. We show that *Yy2* levels can be attenuated in mESC, while still affecting colony formation. *Yy2* depletion did not affect *Oct4* and *Esrrb* levels in our experiments. This may be compatible with the nature of the colonies obtained with sh1*Yy2*, 80% of which were either undifferentiated or consisted of a core of undifferentiated cells. A second explanation is the enhanced expression of these factors upon attenuation of *Yy2* levels (as reported previously [25]) in a relatively diminished number of pluripotent cells, resulting in the observed lack of overall change in gene expression levels (Figure 4). It also remains possible that the effect of *Yy2* depletion on the levels of core pluripotency factors is indeed temporal, as suggested by the absence of effects after 16 h (Figure 4B), or after 7 days (Figure 4A), in contrast to increased levels observed after 48 h [25], in different studies. Independent of the explanation, our results do not provide further proof for the role of core pluripotency factors in *Yy2*-mediated mESC self-renewal. Future studies aimed at understanding the contribution of *Yy2* to the expression of core pluripotency factors will have to consider off-targeting effects of sh*Yy2*, establish appropriate time-courses, and test for genes beyond the restricted set analyzed in our studies.

Although *Zscan4* was not identified as a YY2-binding target in mESC [25], we observed that the attenuation of *Yy2* levels was concomitant with higher *Zscan4* expression. Surprisingly, however, *Zscan4* levels in preimplantation embryos seemed indifferent to *Yy2* depletion. However, given the weak effect of *Yy2* siRNAs in two-cell embryos (Figure 2B), an effect of YY2 on *Zscan4* levels cannot be excluded at this point. Temporal differences in regulation were also observed for REX1-regulated expression of ERV [17]. This observation or phenomenon could be explained by the presence of cis-regulatory regions specific for either stage, the epigenetic modifications that allow YY2 binding in one cell type but not the other, or the requirement for stage-specific cofactors for binding or regulation.

In addition to so-called core pluripotency factors [12], several genes which are essential for long-term maintenance of mESC in culture are expressed only in a subset of cells [47]. Cells shuttle between high- and low-expressing states and the resulting heterogeneity reflects the presence of subpopulations of cells. The non-uniform expression of *Zfp42*/*Rex1* [48] and *Stella* (*Dppa3*/*Pgc7*) [49] is related to a primed or naive state of pluripotency [50,51]. A small subset of cells expressing *Zscan4* [36] display a two-cell stage embryo signature marked by the activation of MERVL endogenous retrovirus [32] and the expression of *Zscan4* co-regulated genes including *Prame*, *Thoc*, and *Tcstv* [52,53]. Both *Yy2* (Figure 4) and *Rex1* [13] deficiency alter *Zscan4* levels in mESC and two-cell embryos, respectively, raising the possibility of co-regulation of this *locus* by these two factors. We do not know whether they converge at the level of DNA binding or through LSD1 interaction, which is shared by both factors. Attempts to quantitate *Zscan4*-positive cells in *Yy2*- or double *Yy2* + *Rex1*-depleted mouse ES colonies may help to clarify this issue. We entertain the possibility that *Yy2* exerts an essential function in the regulation of the expression of *Zscan4*, whose expression in turn is essential for long-term culture of mESC [36].

## Figures and Tables

**Table 1:** Developmental progress assessment of *Yy2*-depleted preimplantation embryos.

	2-Cell	5–8 Cells	≥Morula	Blastocyst
	Day 2	Day 3	Day 4	Day 5
siControl	104/104	81/92	72/80	21/26
	(100%)	(88.0%)	(90.0%)	(80.8%)
si*Yy2*	113/114	83/101	80/86	58/76
	(99.1%)	(82.2%)	(93.0%)	(76.3%)

Injections of control siRNAs (siControl) and siRNAs against *Yy2* (si*Yy2*) were performed in parallel and embryonic development was monitored daily. The ratio of the number of embryos at the specified stage over the total number of embryos, and the average percentage, is indicated. The day of zygotic microinjection was set as Day 1. Cumulative data of three independent experiments are presented, except for data at Day 5, which corresponds to one and two experiments for siControl and si*Yy2*, respectively. A technical issue for the control condition on Day 5 precluded normal developmental progression; therefore, Day 5 data of this condition are limited to only one experiment (see Table S1).

## Data Availability

The data and materials generated for and described in the current study are available from corresponding authors on request.

## Supplementary Materials

The following are available online at https://www.mdpi.com/article/10.3390/cells10051123/s1, Figure S1: *Yy2* expression during embryonic development, Figure S2: Specificity of si*Yy2* and developmental progression of si*Yy2*-injected embryos, Figure S3: Comparison of gene expression levels in shRNA-treated and non-treated mESC, Table S1: Developmental progress of preimplantation embryos, Table S2: Primers and siRNA used.
