# Supplementary material for: YY2 in Mouse Preimplantation Embryos and in Embryonic Stem Cells
Source: Cells. 2021 May 7;10(5):1123. doi: 10.3390/cells10051123 (PMC8148602; doi:10.3390/cells10051123)
Supplement: Supplementary file 1 [file cells-10-01123-s001.zip › cells-1170830-supplementary/cells-1170830-supplementary figures.pdf]

A

Developmental stage	TPM	Gene EST	Total EST in pool
Oocyte	0	0	19348
Unfertilized Ovum	0	0	20312
Zygote	0	0	28807
Cleavage	181	5	27537
Morula	27	1	36903
Blastocyst	14	1	68210
Egg cylinder	0	0	12123
Gastrula	0	0	29408
Organogenesis	0	0	130865
Fetus	4	3	673862
Neonate	0	0	108168
Juvenile	6	2	286633
Adult	7	8	1035996

B

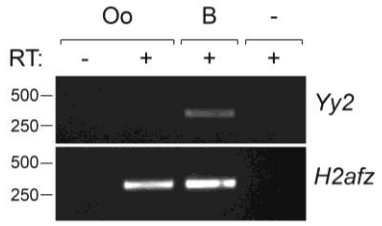

Figure S1. (A) Unigene sequences described in a subset of developmental stages. Transcripts per million (TPM), Gene expressed-sequence tags (EST) and Total EST in pool. **(B)** EtBr stained gel shows PCR products generated with *Yy2* or *H2afz*-specific primers, after reverse transcription of mRNA isolated from a pool of oocytes (Oo) or blastocysts (B). RT refers to Reverse Transcription, a PCR reaction without input (-) is shown to the right. The sizes of Mw markers run alongside are indicated on the left.

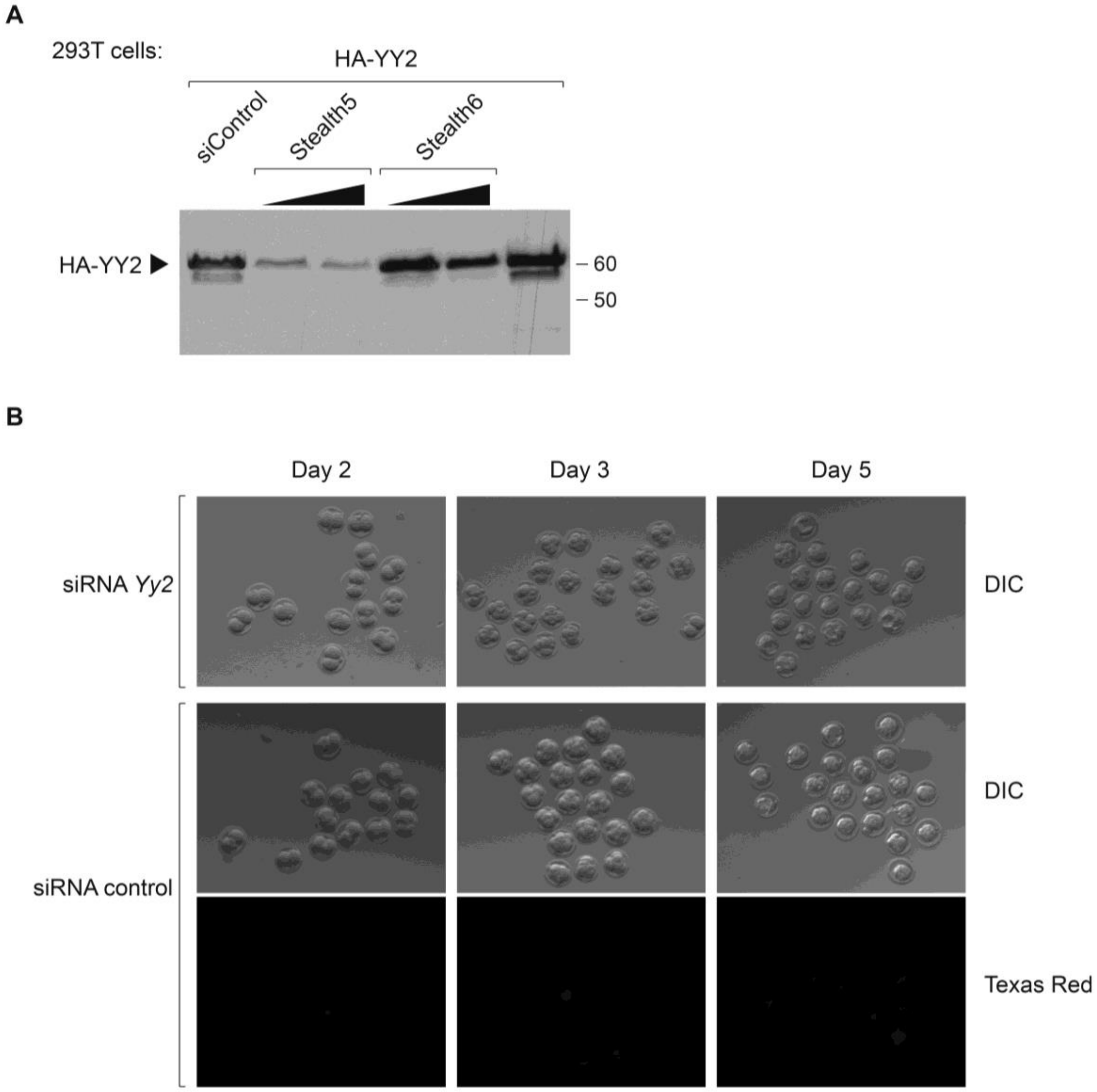

Figure S2. (A) Western blot to detect HA-tagged YY2 in cell lysates from transiently transfected 293T cells using αHA antibody. Figure shows HA-YY2 co-transfected as described (5), with siRNAs (Stealth5 or Stealth6) against *Yy2* or with control siRNAs. Sequences are listed in Supplementary Table 2. Non-transfected 293T cells were included as a negative control (right lane). The ~60 kD band corresponding to HA-YY2 is indicated with an arrowhead on the left. Migration of molecular weight standards is indicated to the right. **(B)** Representative images showing the *in vitro* development of embryos injected on day 1 with Stealth-5 siRNA directed against *Yy2* or siRNA control, together with Dextran Texas Red.

A

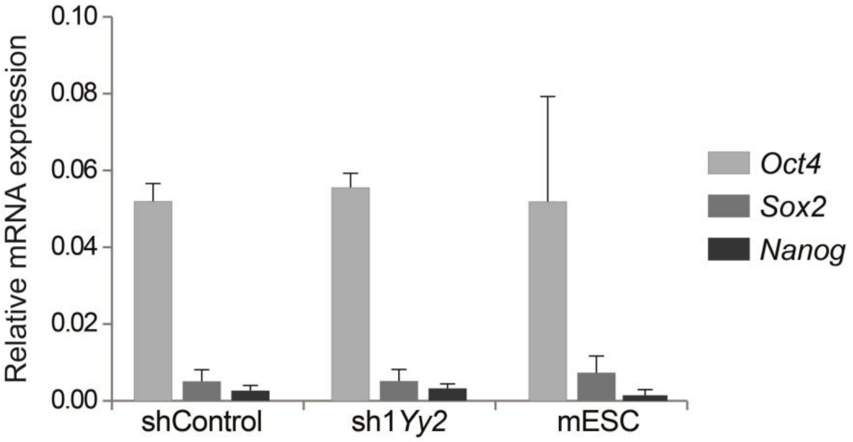

Figure S3. (A) Gene expression of the pluripotency markers *Oct4*, *Sox2* and *Nanog* was measured by RT-qPCR in mESCs, either colonies transfected with shControl-expressing plasmids (shControl) or sh1*Yy*2-expressing plasmids (sh1*Yy2*), or regular non-transfected cells (mESC). The mESC data is reproduced from 24, Figure 1C). Gene expression (three biological replicates) was normalized to *Gapdh* and data is represented as the mean ± standard deviation.
