# Supplementary material for: YY2 in Mouse Preimplantation Embryos and in Embryonic Stem Cells
Source: Cells. 2021 May 7;10(5):1123. doi: 10.3390/cells10051123 (PMC8148602; doi:10.3390/cells10051123)
Supplement: Supplementary file 1 [file cells-10-01123-s001.zip › cells-1170830-supplementary/Supplementary Table 1.pdf]

	2-cell		5-8 cells		≥ morula		blastocyst	
	Day 2		Day 3		Day 4		Day 5	
	Embryos at indicated stage/Total no. of embryos	% embryos at indicated stage	Embryos at indicated stage/Total no. of embryos	% embryos at indicated stage	Embryos at indicated stage/Total no. of embryos	% embryos at indicated stage	Embryos at indicated stage/Total no. of embryos	% embryos at indicated stage
**siControl**								
Experiment 1	22/22	100%	20/22	91%	10/10	100%		
Experiment 2	56/56	100%	39/44	89%	44/44	100%	16/44	36% *
Experiment 3	26/26	100%	22/26	85%	18/26	69%	21/26	80%
Mean		100%		88%		90%		80%
StDev		0%		3%		18%		-
**si*Yy2***								
Experiment 1	25/25	100%	19/25	76%	8/10	80%		
Experiment 2	55/56	98%	36/43	84%	43/43	100%	34/43	79%
Experiment 3	33/33	100%	28/33	85%	29/33	88%	24/33	73%
Mean		99%		82%		89%		76%
StDev		1%		5%		10%		4%
**si*Yy2*+ si*Rex1***								
Experiment 1	26/26	100%	14/14	100%	14/14	100%		
Experiment 2	26/27	96%	14/14	100%	14/14	100%	11/14	79%
Experiment 3	27/28	96%	24/28	86%	21/28	75%	23/28	82%
Mean		98%		95%		92%		80%
StDev		2%		8%		14%		3%

* siControl numbers for this experiment were not included as low embryo progression was due to technical problems.

Table S1. Developmental progress of preimplantation embryos. Developmental progress assessment of preimplantation embryos depleted of either Yy2 or simultaneously depleted of both Yy2 and Rex1. Injections of different siRNAs were performed in parallel and embryonic development was monitored daily. The ratio of the number of embryos at the indicated stage over the total number of embryos, and the average percentage is indicated. 3 independent experiments are shown. Zygotic microinjection day with siRNAs was set as Day 1.
