# Supplementary material for: YY2 in Mouse Preimplantation Embryos and in Embryonic Stem Cells
Source: Cells. 2021 May 7;10(5):1123. doi: 10.3390/cells10051123 (PMC8148602; doi:10.3390/cells10051123)
Supplement: Supplementary file 1 [file cells-10-01123-s001.zip › cells-1170830-supplementary/Supplementary Table 2.pdf]

Table S2. Primers and siRNA used.

A) RT-PCR/RT-qPCR OLIGO SEQUENCES

**Gene code**	**Use** *(except indicated, used for both PCR and qPCR)*	**Forward primer (5'→ 3´)**	**Reverse primer (5'→ 3´)**
Yy2	* RT-PCR	GCATGAGACCCAAAAAGCCAAAAGGAGA	CAGGCATCAAATGGACACACGAAAGGTT
Yy2	* RT-qPCR	CGTTCAGACACGAGAGGAGG	CGCCAGAAAGCCCGTAAAAG
Yy1		TCGCCTGATCGACCTAGAAC	CATAGGGCCCACGACTTAAA
Rex1		AAGCCGTATCAGTGCACGTTCGAAGGCT	ATGCGTGTATCCCCAGTGCCTCTGTCAT
Zscan4		GAGATTCATGGAGAGTCTGACTGATGAGT[illegible]	GCTGTTGTTTCAAAAGCTTGATGACTTC
MERVL		TTTCTCAAGGCCCACCAATAGT	GACACCTTTTTTAACTATGCGAGCT
IAP		TCAAGGACAGGGTATTGTTG	TCGGGTGAGTCTTTCTGGTAC
Oct4		GGCGTTCTCTTTGGAAAGGTGTTC	CTCGAACCACATCCTTCTCT
Nanog		CACCCACCCATGCTAGTCTT	ACCCTCAAACTCCTGGTCCT
Sox2		TAGAGCTAGACTCCGGGCGATGA	TTGCCTTAAACAAGACCACGAAA
Esrrb		TGAGATCACCAAACGGAGGC	AATCCAGCCGTCGCTTGTAC
H2afZ		CGTATCACCCCTCGTCACTT	AAGCCTCCAACTTGCTCAAA
Gapdh		CATACCAGGAAATGAGCTTG	ATGACATCAAGAAGGTGGTG

B) SEQUENCES of siRNAs

**Gene**	**siRNA name**	**Forward primer (5'→ 3´)**	**Reverse primer (5'→ 3´)**
Rex1	si*Rex1*	CGAGUUCCCUUCUCGAGUCUUCUU	AAGAAGACUCGAGAAGGGAACUCG
Yy2	si*Yy2 (Stealth5)*	CCAAGACCUAUAGCAUGCUCUCAUA	UAUGAGAGCAUGCUAUAGGUCUUGG
Yy2	*Stealth6*	CACAGUCAACAAACCUGAAAUCGCA	UGCGAUUUCAGGUUUGUUGACUGUG
